# Experimental evidences for reducing Mg activation energy in high Al-content AlGaN alloy by Mg_Ga_ δ doping in (AlN)_m_/(GaN)_n_ superlattice

**DOI:** 10.1038/srep44223

**Published:** 2017-03-14

**Authors:** Xiao Wang, Wei Wang, Jingli Wang, Hao Wu, Chang Liu

**Affiliations:** 1 Key Laboratory of Artificial Micro- and Nano-structures of Ministry of Education, School of Physics and Technology, Wuhan University, Wuhan 430072, China

## Abstract

P-type doping in high Al-content AlGaN alloys is a main challenge for realizing AlGaN-based deep ultraviolet optoelectronics devices. According to the first-principles calculations, Mg activation energy may be reduced so that a high hole concentration can be obtained by introducing nanoscale (AlN)_5_/(GaN)_1_ superlattice (SL) in Al_0.83_Ga_0.17_N disorder alloy. In this work, experimental evidences were achieved by analyzing Mg doped high Al-content AlGaN alloys and Mg doped AlGaN SLs as well as Mg_Ga_ δ doped AlGaN SLs. Mg acceptor activation energy was significantly reduced from 0.378 to 0.331 eV by using Mg_Ga_ δ doping in SLs instead of traditional doping in alloys. This new process was confirmed to be able to realize high p-type doping in high Al-content AlGaN.

High Al-content AlGaN alloys are ideal materials for deep ultraviolet (DUV)^1^ optoelectronic devices due to their large direct band gaps with operating wavelengths from 364 nm down to 200 nm^2^,^3^,^4^,^5^. The external quantum efficiency (EQE) of AlGaN-based DUV light-emitting diodes (LEDs), however, is as extremely low as 0.1%, which is still a formidable obstacle^6^,^7^. N-type AlGaN can be produced relatively easily^8^,^9^. The very low p-type doping efficiency in AlGaN hinders the further improvement of AlGaN-based DUV LEDs. The difficulty to realize p-type doping is related to the high acceptor activation energy (E_A_), the compensation by nitrogen vacancies, the increased hole scattering, and the limited acceptor solubility^10^,^11^. For the most widely used p-type dopant of Mg, its E_A_ in Al_x_Ga_1−x_N increases monotonically with increasing Al-content from 0.17 eV in GaN to 0.51 eV in AlN^11^,^12^. This behavior indicates that only a very tiny fraction (~10^−9^) of Mg dopants can be activated in AlN at room temperature ^11^. Therefore, decreasing Mg acceptor activation energy is one of the most challenges in AlGaN-based DUV optoelectronic devices.

Great efforts have been devoted to improve p-type conduction in group-III nitrides^13^,^14^,^15^,^16^,^17^,^18^,^19^,^20^. Different from suppressing the charge separation effect in InGaN-based devices, polarization doping has been applied to increase the hole concentration in AlGaN alloys by ionizing Mg acceptor in the polarization field^13^,^14^. Alternative acceptor-donor co-doping and non-equilibrium growth with Mg pulse doping and Mg δ-doping have also been developed to reduce the acceptor activation energy, and thus, increase the hole concentration and enhance the p-type conductivity of AlGaN alloys^15^,^16^,^17^,^18^,^19^,^20^. So far, most experiments were focused on the p-type conductions of GaN and low Al-content AlGaN alloys, but the bottlenecks of the p-type doping in high Al-content AlGaN still remain^20^,^21^,^22^.

Many works were concentrated on the doping in the superlattices (SLs)^23^,^24^,^25^,^26^,^27^,^28^,^29^, where a periodic oscillation of the valence band edge was created by the valence band discontinuity and innate polarization fields in Mg-doped AlGaN/GaN SLs, resulting in the accumulation of holes near the valance band edge close to the Fermi energy forming the so-called two-dimensional (2D) hole gases. In our previous work, we have used Si_Ga_ δ doping in Al_0.6_Ga_0.4_N alloys to increase the n-type carrier density^30^. Recent theoretical works predicted that the nanoscale (AlN)_m_/(GaN)_n_ (m > n) SL could convert the valence-band maximum (VBM) from the crystal-field split-off hole to heavy hole band, leading to the increase of the transverse electric (TE) polarized light emission efficiency^31^,^32^. The influence of the nearest and next-nearest (NN) atoms on Mg electronic structures in nanoscale (AlN)_5_/(GaN)_1_ SL substitution for Al_0.83_Ga_0.17_N disorder alloy was theoretically investigated by Zhong *et al*.^1^. The results showed that the E_A_ decreases if the NN Ga atom number increased and the Mg-centered tetrahedron volume decreased. In this way, the Mg acceptor activation energy can significantly be reduced to 0.26 eV, very close to that of GaN, in (AlN)_5_/(GaN)_1_ SL by Mg_Ga_ δ-doping^1^. Recently, improved p-type conductivity was achieved in multidimensional Al_0.63_Ga_0.37_N/Al_0.51_Ga_0.49_N SLs^33^. In this work, we use Mg_Ga_ δ doping in (AlN)_m_/(GaN)_n_ SLs to study Mg acceptor activation energy, aiming to find a proper way to minimize it in high Al-content AlGaN.

## Methods

**Figure 1:**
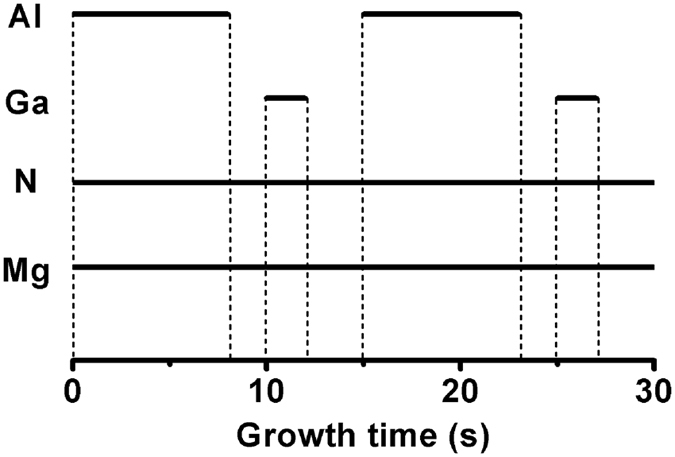
Two loops of the growth process of sample D5. The solid and blank lines indicates open and close of the source shutters, respectively.

In this work, traditional Mg doped AlGaN alloys (in which Al, Ga, Mg and N atoms arrived at the substrate at the same time), Mg doped AlGaN SLs and Mg_Ga_ δ doped AlGaN SLs were grown on c-plane sapphire substrates by using the radio-frequency plasma-assisted molecular beam epitaxy system (rf-MBE, SVTA 35-V-2). The growth details are shown in Table 1 Sample D1 and D2 were Mg doped AlGaN alloys with traditional doping method. Mg doping was continuously carried out for 30 min. Sample D3 and D4 were Mg doped AlGaN SLs. Mg doping was continuously performed for 10 s at each cycle and the total deposition lasted for 180 cycles. Hence the real Mg doping time was also 30 min. Sample D5 and D6 were Mg_Ga_ δ doped AlGaN SLs with a cycle period of 15 s for 180 cycles. Although the cycle period of sample D5 and D6 was 15 s, the effective growth time in a cycle was still kept as 10 s and the total effective growth time was also 30 min. For sample D5, the growth process consisted of two loops as shown in Fig. 1. During the growth of AlGaN thin films, the nitrogen flow rate was set at 2.65 sccm under 375 W rf-plasma power. Prior to the growth, nitridation was performed at 810 °C for 10 min under 500 W rf-plasma power with a nitrogen flow rate of 2.65 sccm. AlGaN films were examined by high-resolution x-ray diffraction (HRXRD, Bede D1) and high-resolution transmission electron microscopy (HRTEM, JEOL JEM 2010 FEF UHR). Ni/Au electrodes (15 nm Ni and 50 nm Au) were made by thermal evaporation with templates of 150 × 150 μm^2^ in area. Current versus voltage (I-V) characteristics were measured by using a semiconductor device analyzer (Keithley 4200, Keithley Instruments).

## Results

**Figure 2:**
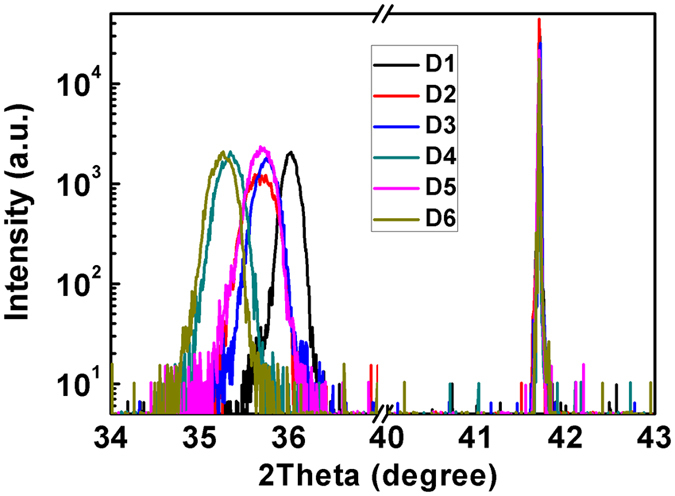
XRD spectra of AlGaN films.

Figure 2 shows XRD patterns of AlGaN films grown on sapphire substrates. AlGaN (0002) peaks were found between the GaN (0002) peak at 34.543° and AlN (0002) peak at 36.033°. The Al_2_O_3_ (0006) peaks were normalized at 41.700°. The full widths at half maximum (FWHM) of the Mg-doped AlGaN (0002) peaks were around 800 arcsec in alloys and 1000 arcsec in SLs. AlGaN peaks were fitted with Gauss model to get more accurate peak information. According to the alloy crystal parameter formula 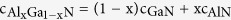 and Bragg’s law 2d_hkl_sinθ = nλ as well as hexagonal interplanar distance formula 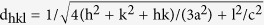, the compositions of Al_x_Ga_1−x_N thin films were determined by using standard crystal parameter c_GaN_ of 0.5189 nm and c_AIN_ of 0.4981 nm. In this way, the compositions of all samples from D1 to D6 were Al_0.97_Ga_0.03_N, Al_0.75_Ga_0.25_N, Al_0.79_Ga_0.21_N, Al_0.53_Ga_0.47_N, Al_0.76_Ga_0.24_N and Al_0.47_Ga_0.53_N, respectively. The compositions of (AlN)_m_/(GaN)_n_ in D3 and D5 nearly matched to the designed value of 4:1, while those in D4 and D6 nearly approached to the designed 1:1.

**Figure 3:**
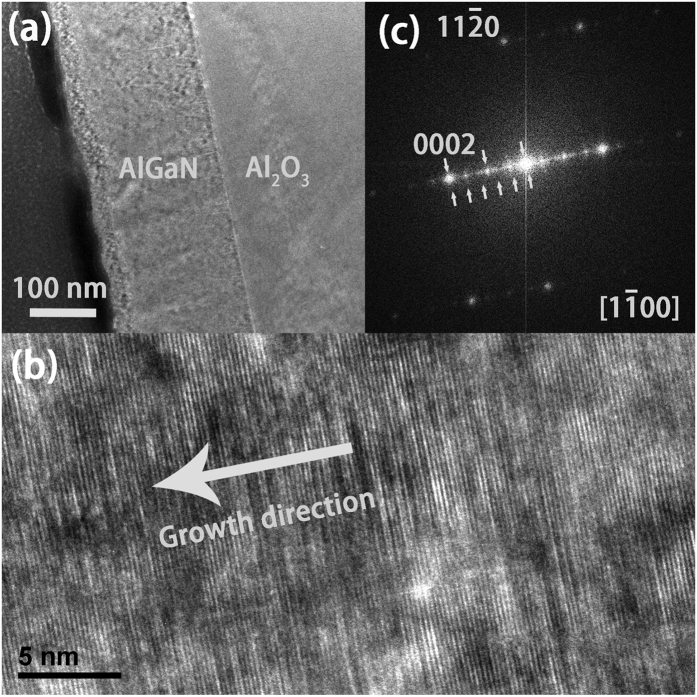
(**a**) Cross-sectional HRTEM image of AlGaN/Al_2_O_3_ in sample D5. (**b**) The magnified HRTEM image of AlGaN superlattices in sample D5. (**c**) FFT image of (**b**).

As XRD results reveal only the macro compositions of the AlGaN films, the (AlN)_m_/(GaN)_n_ SLs were confirmed by HRTEM results. Figure 3(a) shows the cross-sectional HRTEM image of AlGaN SLs grown on sapphire in sample D5. The total thickness of AlGaN SLs were about 210 nm. The magnified HRTEM image of AlGaN SLs and the corresponding FFT image are shown in Fig. 3(b) and (c). The growth direction of AlGaN SLs on sapphire was [0002], in agreement with the XRD results. As shown in Fig. 3(c), five extra diffraction spots were obtained along [0002] axis in one unit. The four quinquesection spots were attributed to the (AlN)_4_/(GaN)_1_ SLs, indicating that the monolayer SL structure was achieved and NN Ga atom number increased in sample D5. The one bisection spot might be caused by the dislocations. Hence sample D5 was measured to be (AlN)_4_/(GaN)_1_ superlattices, achieving the designed (AlN)_m_/(GaN)_n_ SLs structure with increased NN Ga atom number.

**Figure 4:**
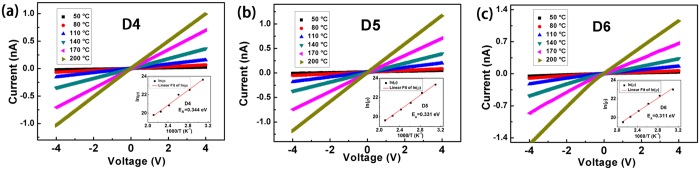
I-V characteristics of (**a**) sample D4, (**b**) sample D5 and (**c**) sample D6 at different temperatures. The insets show the corresponding Arrhenius plots of the resistivity versus temperature. The ionization energy of Mg in AlGaN in sample D4, D5 and D6 was determined to be 0.344, 0.331 and 0.311 eV, respectively.

Figure 4 shows the I-V characteristics of sample D4, D5 and D6 at different temperatures. The linear I-V behavior indicated the Ohmic contacts between Ni/Au electrodes and AlGaN films. The good ohmic behavior could be attributed to the formation of p-type NiO^34^. As for sample D5, the resistances were deduced to be 13.67 GΩ, 5.776 GΩ, 2.090 GΩ, 1.042 GΩ, 547.8 MΩ and 340.3 MΩ at temperatures of 50, 80, 110, 140, 170 and 200 °C, respectively. The insets show the corresponding Arrhenius plot of the resistivity (*ρ*) versus temperature (T). Since 
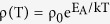
, the ionization energy (E_A_) of Mg in Al_x_Ga_1−x_N in sample D5 was fitted to be 0.331 eV as shown in Fig. 4(b). The same procedures were also performed for other samples. As for the asymmetry I-V curves as shown in Fig. 4(c), E_A_ was calculated separately under positive and negative voltages and then made of an average. The E_A_ for samples D1-D6 were 0.386, 0.378, 0.358, 0.344, 0.331, and 0.311 eV, respectively. The highest E_A_ as 0.386 eV was in sample D1 and the lowest E_A_ as 0.311 eV lied in sample D6, in match with calculated results that high E_A_ in high Al component alloys, and low E_A_ in low Al component SLs.

**Figure 5:**
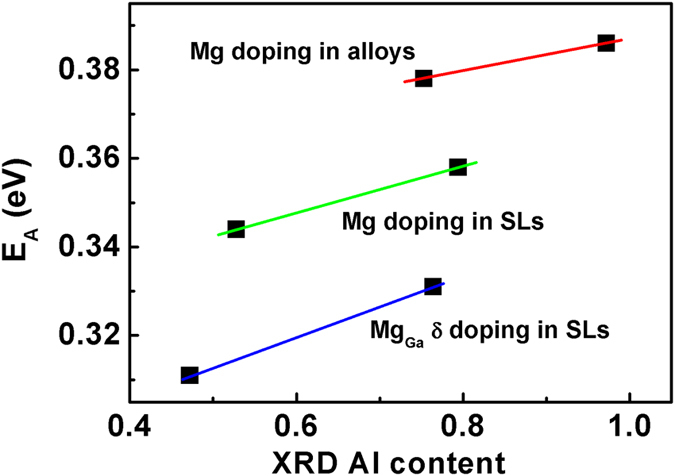
The dependence of E_A_ on Al composition in Al_x_Ga_1−x_N with three doping methods.

Figure 5 shows the dependence of E_A_ on Al composition in AlGaN with three doping methods. Obviously, the E_A_ reaches the lower values in Mg_Ga_ δ doped AlGaN SLs, the medium in Mg doped SLs and the higher in traditional Mg doped AlGaN alloys. As for Al content around 0.8, the E_A_ decreases from 0.378 to 0.358, then to 0.331 eV by the three methods in turn as Mg doped AlGaN alloys, Mg doped SLs and Mg_Ga_ δ doped SLs. Hence, Mg acceptor activation energy can be significantly reduced from 0.378 to 0.331 eV by using Mg_Ga_ δ doping in (AlN)_4_/(GaN)_1_ SLs instead of traditional Mg doping in Al_0.8_Ga_0.2_N alloys. The difference between the theoretical value (0.26 eV) and the experimental one (0.331 eV) is attributed to two reasons: firstly, the macro function of Mg activation energy in Ga and Al was measured in this experiment while only the Mg activation energy in Ga was calculated to be 0.26 eV; secondly, the as-grown (AlN)_4_/(GaN)_1_ SLs were not perfect single crystal with dislocations which might affect the Mg activation energy. Therefore, we have experimentally proved the theoretical prediction that Mg acceptor activation energy can be significantly decreased in (AlN)_m_/(GaN)_n_ SL^1^.

## Conclusions

In conclusion, we have systematically studied Mg doping in high Al-content AlGaN by using different doping methods. For high Al-content AlGaN, Mg acceptor activation energy can be significantly reduced from 0.378 to 0.331 eV by using Mg_Ga_ δ doping in (AlN)_4_/(GaN)_1_ SLs instead of traditional Mg doping in Al_0.8_Ga_0.2_N alloys. Our experimental study verifies the prediction of the first-principles calculations^1^, and provides potential applications in AlGaN-based DUV optoelectronic devices.

## Additional Information

**How to cite this article:** Wang, X. *et al*. Experimental evidences for reducing Mg activation energy in high Al-content AlGaN alloy by Mg_Ga_ δ doping in (AlN)_m_/(GaN)_n_ superlattice. *Sci. Rep.* **7**, 44223; doi: 10.1038/srep44223 (2017).

**Publisher's note:** Springer Nature remains neutral with regard to jurisdictional claims in published maps and institutional affiliations.

## Figures and Tables

**Table 1:** The growth details of all the samples from D1 to D6.

Sample ID	T_Al_ (°C)	T_Ga_ (°C)	T_substrate_ (°C)	T_Mg_ (°C)	t_Al_ (s)	t_Gap_ (s)	t_Ga_ (s)	t_Gap_ (s)
D1	1260	960	795	340	—	—	—	—
D2	1250	970			—	—	—	—
D3	1260	960			8	—	2	—
D4	1260	960			5	—	5	—
D5	1260	960			8	2	2	3
D6	1260	960			5	2	5	3

Sample D1 and D2 were traditional Mg doped alloys. Sample D3 and D4 were Mg doped SLs. Sample D5 and D6 were Mg_Ga_ δ doped SLs. The time “t” inside is the open time in one single loop. “−” means 0 s.
